# CRISPR/Cas9-Mediated Genomic Deletion of the *Beta-1*, *4 N-acetylgalactosaminyltransferase 1* Gene in Murine P19 Embryonal Carcinoma Cells Results in Low Sensitivity to Botulinum Neurotoxin Type C

**DOI:** 10.1371/journal.pone.0132363

**Published:** 2015-07-15

**Authors:** Kentaro Tsukamoto, Chikako Ozeki, Tomoko Kohda, Takao Tsuji

**Affiliations:** 1 Department of Microbiology, Fujita Health University School of Medicine, Toyoake, Aichi, Japan; 2 Department of Veterinary Science, Graduate School of Life and Environmental Sciences, Osaka Prefecture University, Osaka, Japan; Institute Pasteur, FRANCE

## Abstract

Botulinum neurotoxins produced by *Clostridium botulinum* cause flaccid paralysis by inhibiting neurotransmitter release at peripheral nerve terminals. Previously, we found that neurons derived from the murine P19 embryonal carcinoma cell line exhibited high sensitivity to botulinum neurotoxin type C. In order to prove the utility of P19 cells for the study of the intracellular mechanism of botulinum neurotoxins, ganglioside-knockout neurons were generated by deletion of the gene encoding beta-1,4 N-acetylgalactosaminyltransferase 1 in P19 cells using the clustered regularly interspaced short palindromic repeats combined with Cas9 (CRISPR/Cas9) system. By using this system, knockout cells could be generated more easily than with previous methods. The sensitivity of the generated beta-1,4 N-acetylgalactosaminyltransferase 1-depleted P19 neurons to botulinum neurotoxin type C was decreased considerably, and the exogenous addition of the gangliosides GD1a, GD1b, and GT1b restored the susceptibility of P19 cells to botulinum neurotoxin type C. In particular, addition of a mixture of these three ganglioside more effectively recovered the sensitivity of knockout cells compared to independent addition of GD1a, GD1b, or GT1b. Consequently, the genome-edited P19 cells generated by the CRISPR/Cas9 system were useful for identifying and defining the intracellular molecules involved in the toxic action of botulinum neurotoxins.

## Introduction

Botulinum neurotoxins (BoNTs), which are produced by the bacteria *Clostridium botulinum*, are some of the most potent natural toxins. BoNTs are categorized into 7 serologically distinct groups (serotypes A through G); serotypes A, B, E, and F cause botulism in humans and types C and D cause botulism in birds and other animals [1]. All BoNTs cause flaccid paralysis by inhibiting the release of acetylcholine from peripheral neuromuscular junctions [2].

BoNT type C (BoNT/C) is synthesized as a single-chain polypeptide (approximately 150 kDa) that is proteolytically activated into a light chain (50 kDa) and a heavy chain (100 kDa). The light chain intracellularly cleaves synaptosomal-associated protein of 25 kDa (SNAP-25) and syntaxin-1 [3,4], which are both members of the soluble *N*-ethylmaleimide-sensitive factor attachment protein receptor (SNARE) protein family, by acting as a zinc-dependent protease. Cleavage of SNARE proteins, regardless of type, inhibits neurotransmitter release, because their assembly is required for neuroexocytosis. On the other hand, the heavy chain comprises two functional domains: an N-terminal half that is involved in translocation of the light chain into the cytosol, and a C-terminal half (H_C_) that is involved in binding to specific receptors [5]. All serotypes of BoNT, except for BoNT/C, recognize both gangliosides and synaptic vesicle proteins as receptors for entering neuronal cells [6–11]. Gangliosides, but not synaptic vesicle proteins, are predicted to play a significant role in the recognition of neuronal cells by BoNT/C, because BoNT/C toxicity is decreased considerably in ganglioside-deficient mice [12]. Moreover, recent studies revealed that BoNT/C interacts with gangliosides at two binding sites, termed the ganglioside-binding pocket 2 (GBP-2) and sialic acid binding site 1 (Sia-1), and their preferences for gangliosides are different [13,14].

Cultured primary neurons from target molecule-deficient mice have often been used to identify the receptors and investigate the intracellular mechanism of BoNTs, because such neurons are highly sensitive to BoNTs [7,8,12]. However, generating knockout mice is costly and time-consuming. Furthermore using cultured primary neurons is problematic due to ethical considerations about the quantity of mice required, the low efficiency of gene transfection, and difficulties in preparing a suitably large number of cells. To solve these problems, we previously identified a new cell line of murine P19 cells that showed high sensitivity to BoNTs [15]. P19 cells are a mouse-derived embryonal carcinoma cell line that is able to differentiate into neuronal cells [16]. In comparison with neurons differentiated from embryonic stem cells, neuronal cells derived from P19 cells can be prepared more easily and require a shorter period of time before used in toxicity assays. Thus, it is easier to use P19 cells to examine the functions of intracellular molecules that contribute to the toxic action of BoNTs.

In this study, to prove the utility of P19 cells, we attempted to generate ganglioside-knockout neurons by deleting the gene encoding beta-1,4 N-acetylgalactosaminyltransferase 1 (GalNAc-T) using the clustered regularly interspaced short palindromic repeats combined with Cas9 (CRISPR/Cas9) system. The CRISPR/Cas9 system is a recently developed genome-editing technology for generating null mutation of a targeted gene in cell lines [17]. We then evaluated the susceptibility of the generated ganglioside-deficient P19 neurons to BoNT/C. We successfully established P19 cells deficient in gangliosides exhibiting low sensitivity to BoNT/C, and we demonstrated that these genome-edited P19 cells could serve as a new cell-based approach to study the toxic action of BoNTs.

## Materials and Methods

### Materials

The P19 embryonal carcinoma cell line (ATCC, No. CRL-1825) was provided by Dr. Ohkawara (Mie University, Mie, Japan). 1-Palmitoyl-2-oleoyl-sn-glycero-3-phosphocholine (POPC) was purchased from Avanti Polar Lipids (Alabaster, AL). The gangliosides GM1a, GD1a, GD1b, and GT1b were obtained from Sigma-Aldrich (St. Louis, MO), while GD2 and GQ1b were purchased from Calbiochem (La Jolla, CA). *C*. *botulinum* type C strain CB-19 was used for the purification of neurotoxin. BoNT/C was purified according to the method reported previously [18]. Preparation of the mouse monoclonal antibody against GT1b (anti-GT1b mAb) was described previously [19].

### Preparation of fluorescently labeled H_C_/C

Previously described methods were used to express and purify recombinant H_C_ from BoNT/C (H_C_/C: residues E863–E1291) [12]. Briefly, a DNA fragment encoding H_C_/C was cloned into a pET-30a vector to create a His-tag fusion protein. H_C_/C fusion protein was expressed in *Escherichia coli* BL21-CodonPlus (DE3)-RIL, subsequently purified using HisTrap HP (GE Healthcare, Piscataway, NJ) and RESOURCE Q (GE Healthcare) columns. The purified H_C_/C was labeled with Alexa Fluor 488 according to the instructions in the Alexa Fluor 488 Protein Labeling Kit (Life Technologies, Carlsbad, CA).

### Surface plasmon resonance

POPC (5 μmol) in chloroform was mixed with 1 mol % ganglioside GM1a, GD2, GD1a, GD1b, GT1b, or GQ1b in a chloroform/methanol mixture (1:1 volume). The gangliosides were dried to a thin film under nitrogen gas using an evaporator. An additional 0.5 mL of phosphate-buffered saline (PBS) was added by vortexing for 3 min to hydrate the lipid film, yielding a 10 mM solution with respect to POPC. After 5 cycles of freezing and thawing, the suspension was extruded 20 times through a 50-nm filter membrane. Liposomes were also prepared from POPC alone as a negative control. Liposome immobilization on an L1 sensor chip (GE Healthcare) and all surface plasmon resonance (SPR) measurements were performed using a Biacore 2000 system (GE Healthcare). The liposomes (2 mM solution with respect to POPC) were injected at 2 μL/min for 30 min following a 5-min injection of 40 mM octylglucoside at 5 μL/min to wash the surface of the chip. Baseline increased by 6000–8000 RU after this step. The lipid bilayer was then washed for 1 min with 10 mM NaOH at 100 μL/min. Bovine serum albumin (BSA, 0.1 mg/mL; Sigma-Aldrich) was injected onto the sensor chip at 5 μL/min for 5 min to block nonspecific binding. Interactions between BSA and the chip surface did not result in a change of >200 RU. Next, the immobilized sensor chip was primed with HBS-N buffer (GE Healthcare). H_C_/C (15.6, 31.3, 62.5, 125, 250, or 500 nM) in HBS-N buffer was injected for 2 min at 30 μL/min. After 2 min of dissociation with the buffer, a 1 min injection of 1 mM NaOH was applied to regenerate the chip and remove any residual H_C_ on the surface, which was replaced with HBS-N in later subsequent cycles. A sensorgram for the binding of POPC alone was subtracted from sensorgrams as nonspecific binding for the binding of POPC containing gangliosides. Kinetic parameters were measured using BIAevaluation ver. 4.1 Software (GE Healthcare).

### Cell culture and neuronal differentiation

P19 embryonal carcinoma cells were cultured in α-MEM (Life Technologies) with 10% fetal calf serum (BioWest, Nuaillé, France), GlutaMAX (Life Technologies), and penicillin-streptomycin mixture (Life Technologies) (*α*-MEM/10% fetal calf serum) in a humidified 5% CO_2_ atmosphere at 37°C. For neural differentiation, P19 cells were suspended in *α*-MEM/10% fetal calf serum supplemented with 500 nM all-*trans* retinoic acid (ATRA; Sigma-Aldrich) and transferred to a Corning Ultra-low attachment culture dish (Sigma-Aldrich). After 4 days ATRA treatment, cell aggregates were washed with PBS, trypsinized, plated onto a poly-D-lysine-coated plates (Becton Dickinson, Franklin Lakes, NJ) or Poly-D-Lysine Cellware 4-Well CultureSlide (Becton Dickinson) in the absence of ATRA to promote neurodifferentiation. The medium was replaced with Neurobasal medium (Life Technologies) supplemented with B-27 (Life Technologies) (Neurobasal/B-27) 4 h after seeding.

### GalNAc-T or alpha-2,8-sialyltransferase knockdown by siRNA treatment

The following Stealth RNAi siRNA duplex oligonucleotides were purchased from Life Technologies: B4galnt1-MSS247218 (for GalNAc-T, 5′-ACCCACACGUGGAGCACUACUUCAU-3′) and St8sia1-MSS277062 (for alpha-2,8-sialyltransferase [Siat8], 5′-CCUCUGUGAGGAGGUGUCCAUCUAU-3′). Stealth RNAi siRNA Negative Control Med GC Duplex #3 (Life Technologies) was used as a negative control. BLAST was used for all Stealth RNAi siRNA sequences against the murine genome database in order to eliminate cross-silencing with non-target genes. The duplexes were transfected into P19 cells with Lipofectamine RNAiMAX (Life Technologies) when the medium was replaced with Neurobasal/B-27 after seeding onto a poly-D-lysine-coated plate. The Stealth RNAi duplex was added at a concentration of 30 nM with 0.3% Lipofectamine RNAiMAX reagent. The cells were then incubated for 72 h before use in subsequent assays.

### RNA extraction and quantitative RT-PCR

RNA extraction and subsequent quantitative RT-PCR analyses were used to determine GalNAc-T and Siat8 expression levels and the extent of GalNAc-T and Siat8 silencing at the mRNA level at post-transfection 3 days. Cells were trypsinized, harvested from the plate, and then centrifuged. Total RNA was extracted using the ReliaPrep RNA Cell Miniprep System (Promega, Madison, WI) according to the manufacturer’s instructions. The integrity and quantity of each total RNA extract was assessed using an Agilent 2100 Bioanalyzer (Agilent Technologies, Santa Clara, CA). One microgram of total RNA was reverse transcribed into cDNA using a SuperScript VILO cDNA Synthesis Kit (Life Technologies) according to the manufacturer’s instructions. An ABI PRISM 7900HT Sequence Detection System (Life Technologies) was used to perform quantitative real-time PCR. TaqMan Gene Expression Master Mix and TaqMan Gene Expression Assay probes for amplification of murine GalNAc-T (Mm0048649_g1) cDNA or Siat8 (Mm00456915_m1) cDNA were obtained from Life Technologies. As an internal control, TaqMan probes were used to amplify murine glyceraldehyde-3-phosphate dehydrogenase (GAPDH, Mm99999915_g1). The mRNA levels were calculated for each sample using the cycle threshold (C_t_) value and relative gene expression was calculated using the 2^-ΔΔCt^ method, where ΔΔC_t_ = ΔC_target gene_ − ΔC_GAPDH_ [20].

### Flow cytometry

To calculate the binding and uptake of H_C_/C or cholera toxin B subunit (CTB), P19 neurons 2 days after ATRA treatment were treated with or without 100 nM Alexa Fluor 488-labeled H_C_/C or 25 nM Alexa Fluor 647-conjugated CTB (Life Technologies) in Neurobasal/B-27 medium for 30 min at 37°C, and were then washed with PBS 3 times. The washed cells were harvested using an AccuEasy Flow Cytometry Kit (LEAP Biosciences, Palo Alto, CA) according to the manufacturer’s instructions. Cells were analyzed by flow cytometry with a Gallios Flow Cytometer (Beckman Coulter, Brea, CA). At least 5000 events were acquired for each experiment, and the data were processed with Kaluza Flow Analysis Software (Beckman Coulter). The results are the mean of 3 independent experiments.

### Design of the CRISPR/Cas9 vector

**Fig 1:**
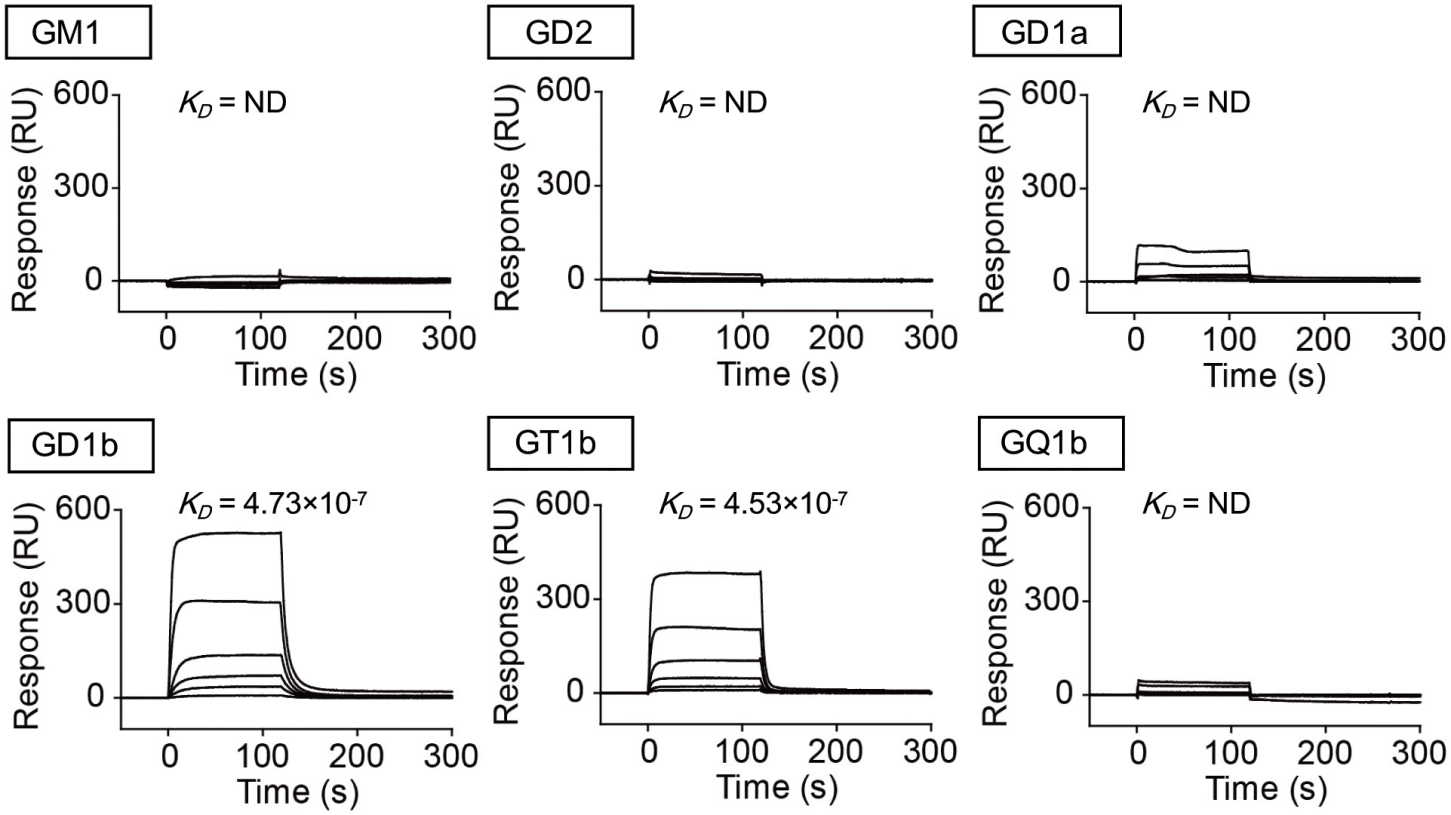
Ganglioside-binding specificity of H_C_/C. Sensorgrams were plotted as the mass of H_C_/C binding (in RU) to immobilized ganglioside (GM1, GD2, GD1a, GD1b, GT1b, and GQ1b)-containing liposomes. A concentration series (15.6, 31.3, 62.5, 125, 250, or 500 nM) of H_C_/C was injected onto immobilized POPC liposomes containing 1% ganglioside. Triplicate curves were fitted globally using BIAevaluation ver. 4.1 software with a 1:1 (Langmuir) binding model to determine the *K*_*D*_ values.

To generate GalNAc-T knockout P19 cells, the *B4galnt1* gene encoding GalNAc-T was deleted using the CRISPR/Cas9 genome editing system and the GeneArt CRISPR Nuclease Vector with an OFP Reporter Kit (Life Technologies) according to the manufacturer’s instructions. Briefly, target-specific oligonucleotides (top strand oligo: 5′-GCATTGTGGTCGCCCCCACAGTTTT-3′, and bottom strand oligo: 5′-TGTGGGGGCGACCACAATGCCGGTG-3′) were synthesized by Life Technologies Japan, Ltd. Annealing both single-stranded oligonucleotides resulted in a double-stranded oligonucleotide with compatible ends for cloning into the GeneArt CRISPR Nuclease Vector that was then ligated into the vector using T4 DNA ligase (Fig 1). The ligated vector was transformed into One Shot TOP10 chemically competent *E*. *coli* (Life Technologies) and the presence of the double-stranded oligonucleotide insert in positive transformants was confirmed by sequencing.

### Generation of GalNAc-T-depleted P19 cells

Undifferentiated P19 cells were transfected with the CRISPR/Cas9 vector and Lipofectamine 3000 (Life Technologies) according to the manufacturer’s instructions. Cells were collected by trypsinization at 48 h after transfection. We screened for the orange fluorescent protein-positive cell clones and sorted them using a MoFlo Astrios Cell Sorter (Beckman Coulter). To check for mutation of the cell clones, a GeneArt Genomic Cleavage Detection Kit (Life Technologies) was used to perform cell lysis, genomic PCR, and detection of cleavage, according to the manufacturer’s instructions. Further, a single cell was isolated by limited dilution of mutated cells in a 96-well plate, then the isolated cell was cultivated and subcloned again: three cells were subcloned. To confirm that both alleles were deleted from chromosomes, the region including the target site was amplified by PCR using the following primer set (forward primer: 5′- AGAGAGGCGGAAGAAAGGAG-3′, and reverse primer: 5′- GCACTGGAGGAAACAAAAGC-3′). PCR products from each of the 3 subcloned cells were ligated into TOPO TA Cloning vectors (Life Technologies). Twenty-four cloned vectors (8 clones each derived from each of 3 subcloned cells) were purified and the target sequence verified by DNA sequencing at Eurofins Genomics, Inc. (Tokyo, Japan).

### Fluorescent staining and confocal microscopy

To observe cell surface GM1 or GT1b, differentiated P19 cells (2 days after ATRA treatment) were fixed in 4% paraformaldehyde/PBS for 15 min at room temperature (RT). Fixed cells were rinsed 3 times with PBS containing 10 mM glycine and then incubated with PBS containing 3% BSA for 30 min at RT and then with 25 nM Alexa Fluor 647-CTB (for GM1) or 5 μg/mL anti-GT1b mAb for 30 min at RT. Cells were washed with PBS/0.1% BSA and incubated with Alexa Fluor 488-conjugated anti-mouse IgG (1:500; Life Technologies) for 30 min at room temperature, washed again with PBS/0.1% BSA, and then incubated with 1 μg/mL DAPI (Life Technologies) for 5 min at RT. Finally, cells were mounted with ProLong Gold Antifade reagent (Life Technologies).

To detect internalized H_C_/C, differentiated P19 cells (2 days after ATRA treatment) were treated with 200 nM Alexa Fluor 488-labeled H_C_/C and NucBlue Live Cell Stain ReadyProbes reagent (1:15, Life Technologies) in Neurobasal/B-27 medium for 20 min at 37°C and washed with ice-cold Hanks' Balanced Salt Solution, no calcium, no magnesium (HBSS, Life Technologies) 3 times. The cells were then treated with CellMask Deep Red Plasma Membrane Stain in ice-cold HBSS (1:700, Life Technologies) for 10 min on ice. The stained cells were fixed in 4% paraformaldehyde/PBS for 15 min on ice. Fixed cells were then rinsed 3 times with PBS containing 10 mM glycine and then mounted with ProLong Gold Antifade reagent.

All images were visualized using an LSM 710 confocal microscope (Zeiss, Oberkochen, Germany) and ZEN2009 software (Zeiss).

### Proteolytic activity of BoNT/C in P19 cells

Differentiated P19 cells (at 2 days after ATRA treatment) were treated with or without BoNT/C (0.04, 0.16, 0.64, 2.5, or 10 nM), and incubated for 30 min at 37°C. After incubation, the cells were washed with PBS, and replaced with the fresh Neurobasal/B-27 medium, then incubated for 18 h in a humidified 5% CO_2_ atmosphere at 37°C. The toxin-treated cells were lysed by scraping into lysis buffer (0.2 M NaCl, 1% Nonidet P-40, 1mM dithiothreitol, 1 mM EDTA) containing a protease inhibitor cocktail (Nacalai, Kyoto, Japan). The cell extract was recovered by centrifugation at 17,000 × *g* for 10 min and combined with an equal volume of 2× NuPAGE sample buffer (Life Technologies) before 10 min of heating at 70°C. Proteins in 5 μg of whole cell extract were separated by SDS-PAGE using a NuPAGE 12% Bis-Tris gel and MOPS-SDS running buffer (Life Technologies) and transferred to a polyvinylidene difluoride membrane using the iBlot Dry Blotting System (Life Technologies), according to the manufacturer’s instructions. After blocking for 1 h at RT with Blocking One (Nacalai), immunodetection was performed using a mouse mAb against syntaxin-1 (1:600, sc-12736; Santa Cruz, Santa Cruz Biotechnology, Inc., CA), SNAP-25 (1:300, sc-20038; Santa Cruz), and GAPDH (1:10000, ab125247; Abcam PLC, Cambridge, UK) in TBST (10 mM Tris, 150 mM NaCl, 0.1% (v/v) Tween 20, pH 7.4) including 0.5% Blocking One for 1 h at RT. After three washes with TBST, a secondary antibody, HRP-conjugated goat anti-mouse IgG (1:2000; Jackson ImmunoResearch, West Grove, PA) in TBST-Blocking One, was added. Blots were washed with TBST after 1 h at RT, and developed using SuperSignal West Dura Extended Duration Substrate (Thermo Scientific, Waltman, WY). The LAS 4000 system (Fujifilm, Tokyo, Japan) was used to visualize signals. Densitometry of the reactive bands was determined using Multi Gauge version 3.0 software (Fujifilm).

To test for proteolytic activity of exogenous gangliosides towards BoNT/C in GalNAc-T-deleted cells, either one of the gangliosides GD1a, GD1b, or GT1b (each 9 μM), or a mixture of these 3 ganglioside (total 9 μM, each 3 μM) was added into the culture of P19 neurons (2 days after ATRA treatment) and incubated for 24 h in a humidified 5% CO_2_ atmosphere at 37°C. After ganglioside treatment, the cells were washed with PBS 3 times, then treated with 4 nM BoNT/C for 30 min at 37°C. After incubation, the cells were washed with PBS, and replaced with the fresh Neurobasal/B-27 medium, then incubated for 18 h in a humidified 5% CO_2_ atmosphere at 37°C. Subsequently, the cells were harvested, lysed, and analyzed by western blotting according to the method described above. One-way ANOVA to test for differences among treatments was performed using GraphPad Prism 5 software and *p* values of less than 0.05 were regarded as significant.

## Results

### Binding specificities of BoNT/C to gangliosides

Gangliosides are required as a receptor component for BoNTs [1]. Using thin layer chromatography, BoNT/C was shown to bind to gangliosides GD1a, GD1b, and GT1b [12, 21]. We investigated the selectivity and specificity of H_C_/C ganglioside recognition using SPR analysis because the affinity of H_C_/C to gangliosides remains unclear (Fig 1). We found that H_C_/C bound to GD1a, GD1b, and GT1b, but not to GM1a, GD2, or GQ1b, and the kinetics parameters (*K*_*D*_ values) of GD1b and GT1b for H_C_/C were almost identical, whereas the *K*_*D*_ values for GD1a could not be calculated because of its low affinity for H_C_/C.

### Knockdown of GalNAc-T or Siat8 by siRNA treatment

**Fig 2:**
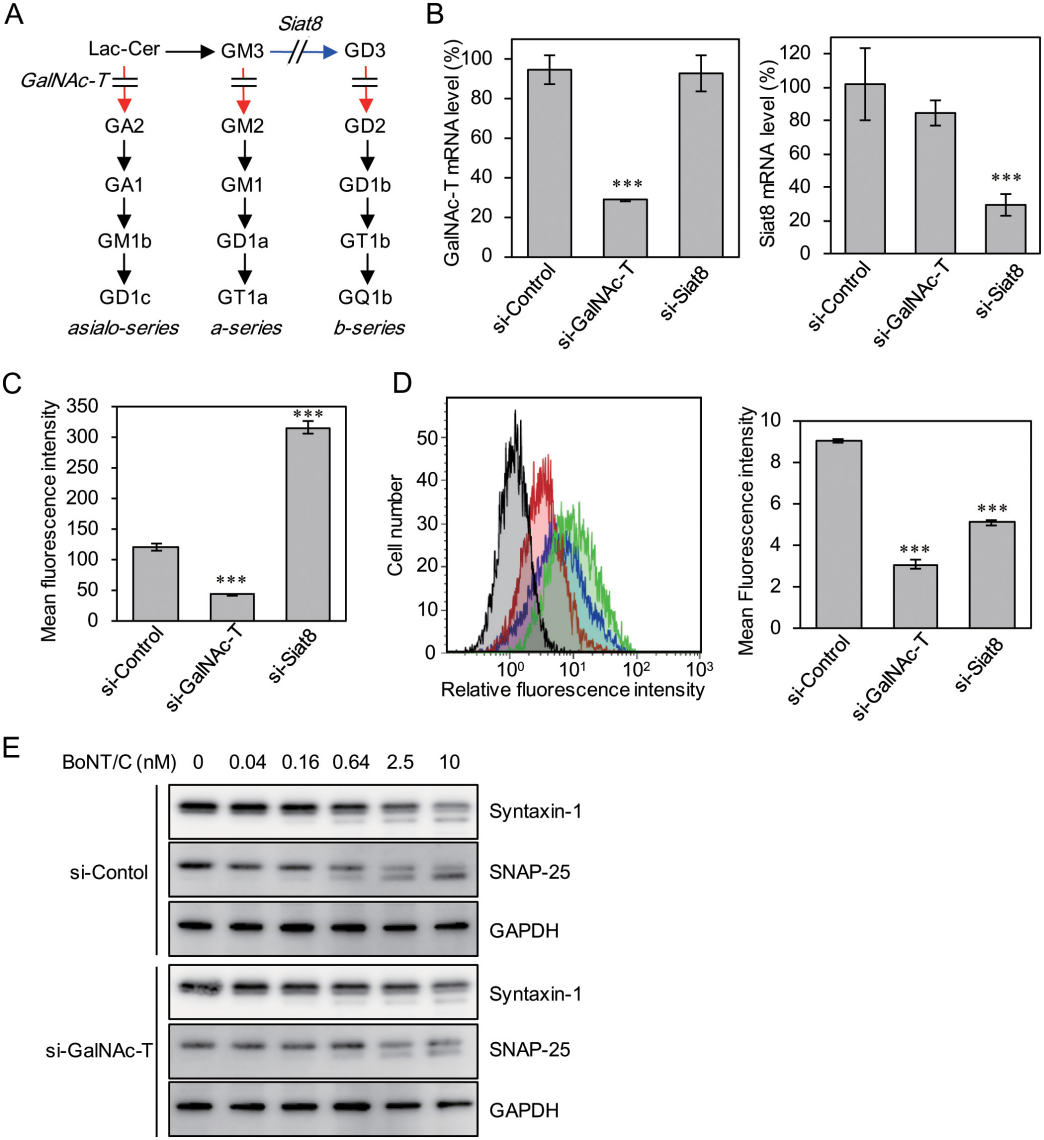
Effects of knockdown of ganglioside synthase genes on binding/entry of H_C_/C into P19 neurons. (A) Synthetic pathway of gangliosides and depletion of ganglioside synthase by siRNA. The red or blue arrow indicates the ganglioside synthesis pathway targeted by GalNAc-T or Siat8, respectively. (B) GalNAc-T (left) and Siat8 (right) mRNA levels of knockdown cells. The mRNA levels obtained from mock-transfected cells were set to 100%. Significance for differences (compared with control) was evaluated by one-way ANOVA (***, *p* < 0.001). (C) Binding and entry of Alexa Fluor 647-CTB into knockdown cells. Mean fluorescence intensities were measured by flow cytometric analysis. Significance for differences (compared with control) was evaluated by one-way ANOVA (***, *p* < 0.001). (D) Flow cytometric analysis for the binding and entry of Alexa Fluor 488-H_C_/C into knockdown cells. Histograms represented the number of cells versus relative fluorescence intensity in the left panel (black: without Alexa Fluor 488-H_C_/C, green: si-Control, red: si-GalNAc-T, blue: si-Siat8). The right panel indicates the mean fluorescence intensities. Significance for differences (compared with control) was evaluated by one-way ANOVA (***, *p* < 0.001). (E) Cleavage of syntaxin-1 and SNAP-25 by BoNT/C in si-RNA-transfected P19 neurons. P19 neurons transfected with control- or GalNAc-T-siRNA were exposed to 0, 0.04, 0.16, 0.64, 2.5, or 10 nM BoNT/C for 30 min at 37°C, then the toxin was removed and cells were incubated for 18 h at 37°C in fresh medium. Cells were then harvested and solubilized, and extracts were subjected to SDS-PAGE and western blotting. The membranes were probed with anti-syntaxin-1, anti-SNAP-25, and anti-GAPDH mAbs followed by an HRP-conjugated goat anti-mouse antibody. GAPDH is shown as an internal control. The reactive bands were visualized with SuperSignal West Dura Extended Duration Substrate.

Gangliosides are synthesized by the ganglioside synthases GalNAc-T and Siat8 (Fig 2A). To examine the function of gangliosides in the uptake of BoNT/C by P19 neurons, we utilized RNAi to knock down these two synthases in P19 cells. Equal amounts of GalNAc-T siRNA, Siat8 siRNA, or negative control siRNA were transfected into P19 cells by lipofection. The mRNA levels of GalNAc-T (Fig 2B, left) and Siat8 (Fig 2B, right) were decreased significantly upon transfection of specific siRNA. We then confirmed the synthesis of gangliosides by measuring CTB binding (Fig 2C). GalNAc-T knockdown resulted in decreased CTB binding. Conversely Siat8 knockdown resulted in increased CTB binding. We next examined the effects of knocking down these two synthases on the binding and uptake of H_C_/C (Fig 2D). The binding and uptake of H_C_/C decreased under both GalNAc-T and Siat8 knockdown, and the fluorescence intensity of H_C_/C following GalNAc-T knockdown was lower than that following Siat8 knockdown. Therefore, the effect of GalNAc-T knockdown on the intracellular activity of BoNT/C was examined next (Fig 2E). Upon BoNT/C treatment, cleavage products of syntaxin-1 and SNAP-25 in GalNAc-T knockdown cells decreased slightly compared to those in control cells.

### Generation of GalNAc-T-knockout P19 neurons

**Fig 3:**
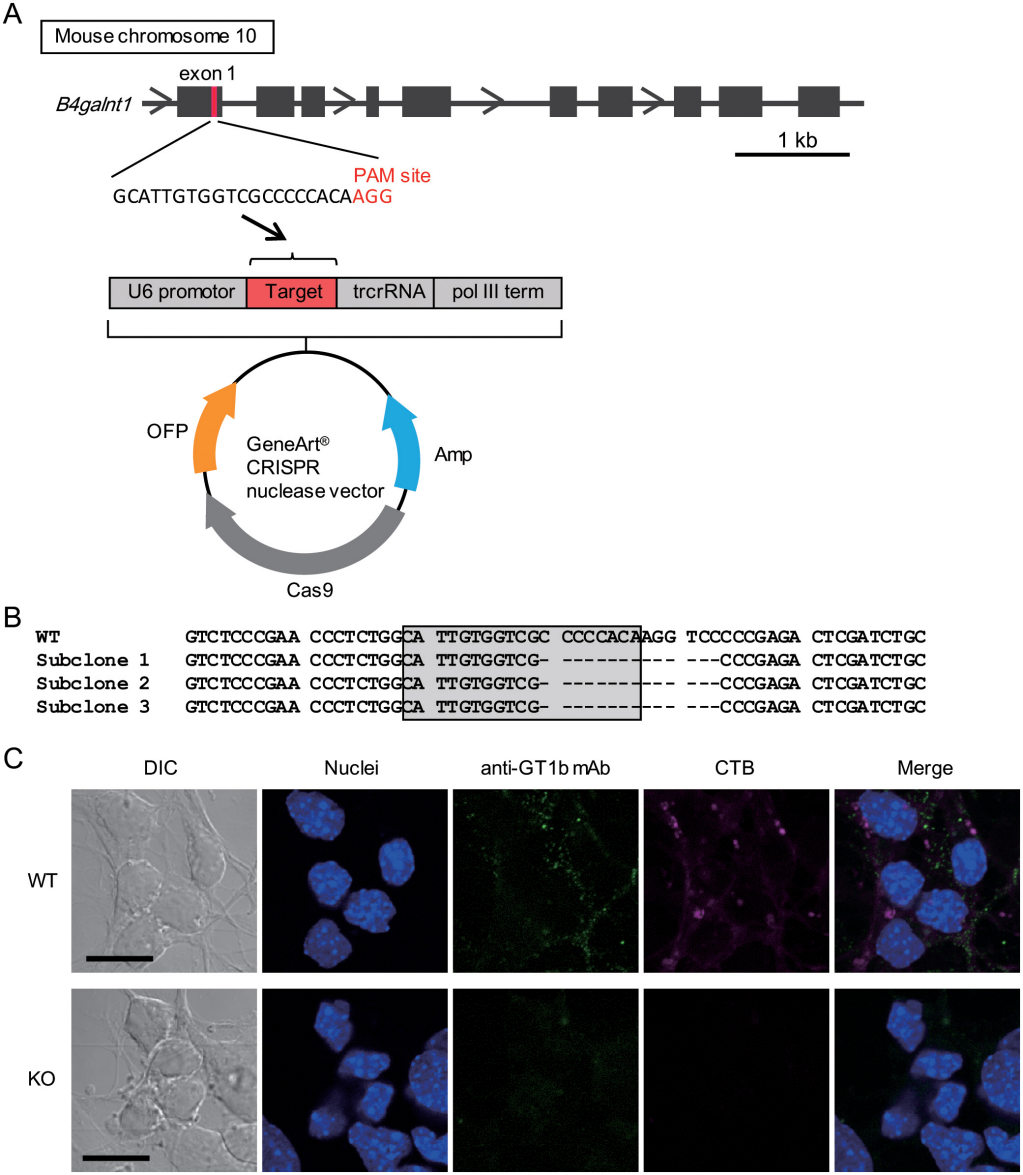
Generation of GalNAc-T-knockout P19 cells. (A) Target sequence derived from the genomic sequence of *B4galnt1* exon 1 was inserted into the GeneArt CRISPR nuclease vector. P19 cells were transfected with the vector using the Lipofectamine 3000 reagent. (B) Sequencing of the neighboring target region in exon 1 of the *B4galnt1* gene of CRIPR/Cas9 vector-transfected P19 cells. Parts of the *B4galnt1* gene from 3 different subcloned cells were amplified by PCR and subcloned into a TOPO cloning vector. Eight TOPO vectors derived from 3 subcloned cells (subclones 1 to 3) were sequenced. All target sites in the vectors contained the 14-nucleotide deletion, and neither other deletions nor original sequence were detected. Gray squares indicate the target sequences. (C) P19 neurons were fixed and stained with an Alexa Fluor 647-labeled CTB (magenta) or anti-GT1b mAb and Alexa Fluor 488-labeled anti-mouse IgG antibody (green). DAPI was used to stain the nuclei (blue). Differential interference contrast (DIC) and fluorescence images were collected using confocal microscopy. Neither CTB, which binds GM1, nor GT1b (using an anti-GT1b mAb) were detected on GalNAc-T-knockout P19 cells. WT and KO indicate wild-type and GalNAc-T-knockout cells, respectively. Scale bars indicate 10 μm.

GalNAc-T knockdown reduced the uptake of H_C_/C, but no notable effect on the proteolytic activity of BoNT/C was observed; therefore, we attempted to generate GalNAc-T-knockout cells utilizing the CRISPR/Cas9 genome editing system. We designed the CRISPR/Cas9 vector to delete the *GalNAc-T* gene and transfected the vector into undifferentiated P19 cells (Fig 3A). The genomic DNA of the cloned cell was analyzed by sequencing. Sequencing of the neighboring target region in exon 1 of the *GalNAc-T* gene revealed that all of the target sites in the subcloned vectors had a 14-nucleotide deletion, and neither other deletions nor the original sequence were observed (Fig 3B). Therefore, we conclude that the cloned cell was derived from a single clone carrying the 14-nucleotide deletion at both alleles and that GalNAc-T-knockout cells were generated. We next examined ganglioside synthesis in P19 neurons of knockout cells using CTB and an anti-GT1b mAb. Both CTB and anti-GT1b mAb were barely detected in GalNAc-T-knockout neurons (Fig 3C).

### Evaluation of off-target effects in GalNAc-T-deficient P19 cells

We evaluated whether GalNAc-T-deficient P19 cells possessed undesired mutations (off-target effects). Previous studies have suggested that genomic sites that have mismatches of fewer than 5 bp with point accepted mutation (PAM) sequences could function as potential off-target sites [22,23]. We looked for potential off-target sites with blastn searches against the mouse genomic database and found that the target sequence had only one potential off-target site (Table 1). To examine whether our GalNAc-T-deficient cells carried off-target mutations, we designed a primer set (forward primer: 5′- CCATACCTGAGGCTAAC-3′, and reverse primer: 5′- TGCTTGCTATTCGCTT-3′) to amplify the potential off-target site, performed PCR amplification, and cloned the PCR products into a TA cloning vector. No in-del mutations in the potential off-target site were observed among the 10 clones verified by DNA sequencing at selected sites. Therefore, we conclude that there were no off-target effects in these GalNAc-T-deficient P19 cells.

**Table 1:** Potential off-target sites in mouse genome.

	Sequence (5'-3')	Locations	Annotations	Mismatches
On-target site	GCATTGTGGTCGCCCCCACAagg	Chromosome 10	B4galnt1	-
Off-target site	**C**C**CC**TGTGGTC**T**CCCCCACAggg	Chromosome 8	Cadherin-13 precursor	4 bp

Genomic sequences of off-target sites with PAM sequences, their locations, annotations, and the number of base pair differences are noted. Bold letters indicate mismatched nucleotides.

### Toxic activity of BoNT/C on GalNAc-T-knockout P19 neurons

**Fig 4:**
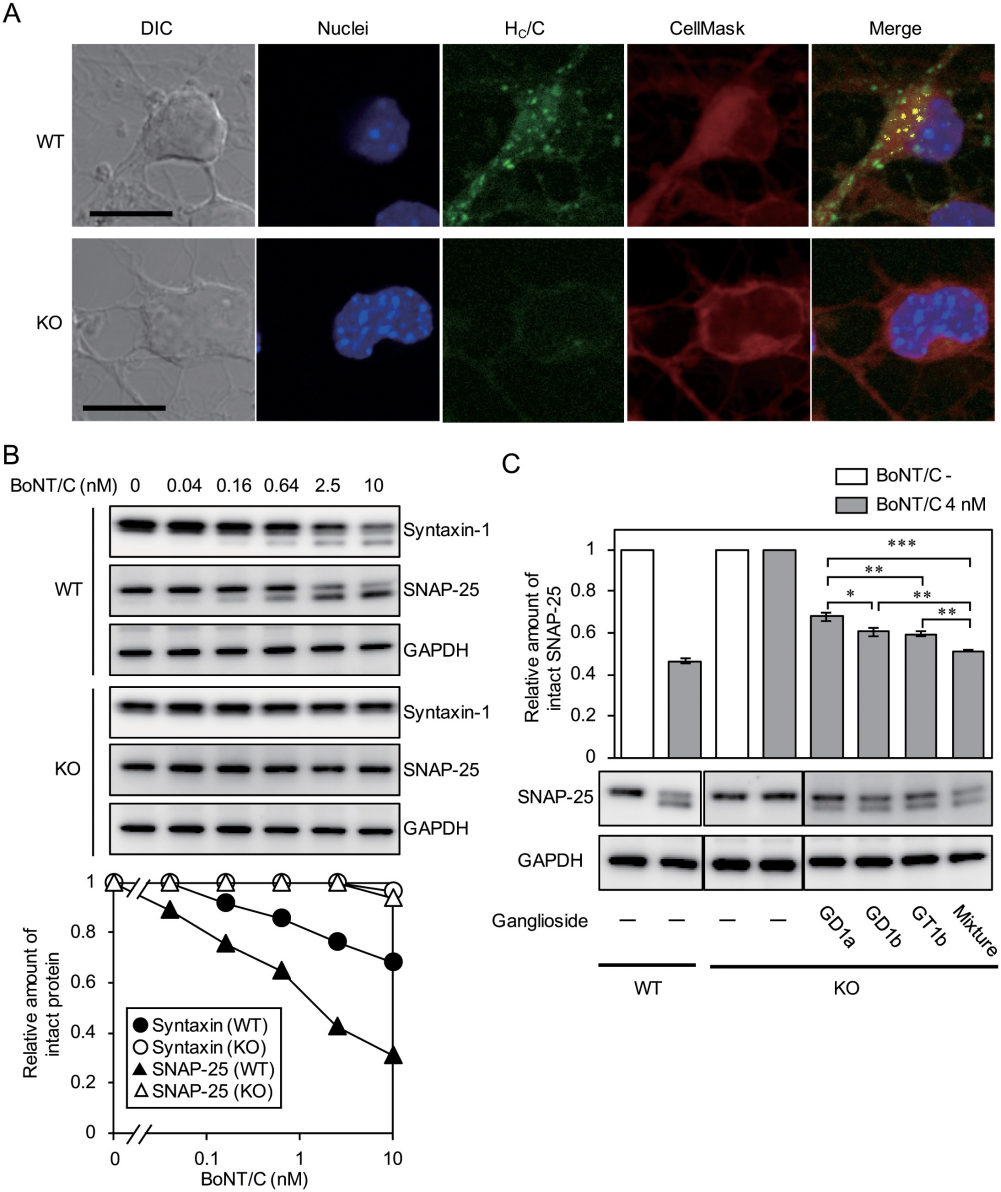
Sensitivity of GalNAc-T-knockout P19 cells to BoNT/C. (A) Uptake of H_C_/C into P19 neurons. P19 neurons were treated with Alexa Fluor 488-labeled H_C_/C (green) and NucBlue Live Cell Stain ReadyProbes reagent for nuclear staining (blue), then plasma membranes were stained with CellMask Deep Red Plasma Membrane Stain (red). Differential interference contrast (DIC) and fluorescence images were collected using confocal microscopy. H_C_/C was detected as punctate signals in P19 neurons derived from wild-type cells, but not in knockout cells. High degrees of H_C_/C and CellMask overlap in merged images are represented in yellow. Scale bars indicate 10 μm. (B) Cleavage of syntaxin-1 and SNAP-25 in P19 neurons by BoNT/C. P19 neurons were exposed to 0.04, 0.16, 0.64, 2.5, or 10 nM BoNT/C for 30 min at 37°C, then the toxin was removed and cells were incubated for 18 h at 37°C in fresh medium. Cells were harvested, solubilized, and extracts were subjected to SDS-PAGE and western blotting. The membranes were probed with anti-GAPDH, anti-syntaxin-1, and anti-SNAP-25 mAbs followed by an HRP-conjugated goat anti-mouse antibody. GAPDH is shown as an internal control. The reactive bands were visualized with SuperSignal West Dura Extended Duration Substrate (upper). The relative amount of intact protein was calculated as a ratio of the relative intensity of each band (intact band/intact + cleaved band). Each point represents the average of three experiments; and because the standard errors for each point were extremely low (<0.01), the error bars are not shown (lower). WT and KO indicate wild-type and GalNAc-T-knockout cells, respectively. Both syntaxin-1 and SNAP-25 were cleaved by BoNT/C in a dose-dependent manner in wild-type cells, but were barely cleaved in knockout cells. (C) Recovery of BoNT/C-induced SNAP-25 cleavage in P19 neurons derived from GalNAc-T-knockout cells by addition of exogenous gangliosides. P19 neurons were treated with the gangliosides GD1a, GD1b, GT1b (each 9 μM), no gangliosides (control), or a mixture of these 3 gangliosides (total 9 μM, each 3 μM) for 24 h. Cells were then exposed to BoNT/C (4 nM) for 30 min. Western blotting analysis was performed at 18 h after removing the toxin for the detection of SNAP-25 cleavage. The relative amount of intact SNAP-25 cleavage was calculated by measuring the intensity of the bands and calculating the ratio of intact to intact + uncleaved bands. Each column represents the average of three independent experiments; error bar indicate the standard error. Significance for differences was evaluated by one-way ANOVA (*, *p* < 0.05; **, *p* < 0.01; ***, *p* < 0.001).

We next investigated the binding, uptake, and proteolytic activity of BoNT/C on the GalNAc-T-knockout neurons. Punctate H_C_/C signals were observed in the cell bodies and neurites of wild-type cells, whereas these signals were not detected in knockout neurons (Fig 4A). The signals of H_C_/C observed in the wild-type cells mainly exist independently of the cell surface marker; however, they were partially co-localized, indicating that the wild-type cells, but not knockout cells, could take up H_C_/C. Moreover, to determine the effect of ganglioside deficiency on BoNT/C activity, wild-type and knockout P19 neurons were treated with BoNT/C, then, cleavage of the syntaxin-1 and SNAP-25 intracellular target proteins of BoNT/C, was detected by western blot analysis (Fig 4B). Intact syntaxin-1 and SNAP-25 levels decreased remarkably in wild-type cells, with the smaller bands representing the cleavage products appearing below those of the intact proteins. Both syntaxin-1 and SNAP-25 in wild-type cells were cleaved in a dose-dependent manner. Conversely, in knockout cells, hydrolysis of syntaxin-1 and SNAP-25 were barely detected. On the basis of these results, we examined whether sensitivity to BoNT/C in knockout cells was restored by the exogenous addition of specific gangliosides (Fig 4C). Restorative effects were found either if we added any one of GD1a, GD1b, or GT1b, but the effect of GD1a addition was lower than that of GD1b or GT1b addition. Particularly, addition of a mixture of all three of these gangliosides effectively recovered the sensitivity of GalNAc-T-knockout cells compared to independent addition of GD1a, GD1b, or GT1b.

## Discussion

BoNTs are the causative agent of botulism and the most lethal biological toxins known. Cell-based analyses are important methods for in-vitro clarification of the intracellular functions and toxicity of BoNTs. Recently, we found that neurons derived from the murine P19 embryonal carcinoma cell line possessed high sensitivity to BoNT/C. In order to further validate the utility of P19 cells, we transfected siRNA into P19 cells and established GalNAc-T-knockout cells using genome-editing technology.

We first confirmed the binding specificity of BoNT/C for gangliosides with SPR analysis. BoNT/C exhibited similar binding affinity for gangliosides GD1b and GT1b, while the affinity of GD1a for H_C_/C was lower than that of GD1b or GT1b. The binding affinities of BoNT/C for gangliosides are first reported in the present study, although its binding specificities had been analyzed previously using a thin layer chromatography overlay assay [12,24]. Following on SPR analysis results, we next examined the effect of knocking down two kinds of ganglioside synthase, GalNAc-T and Siat8, to determine whether gangliosides GD1a, GD1b, and GT1b are able to mediate the entry of BoNT/C into P19 cells. GalNAc-T knockdown had a greater effect on the uptake of BoNT/C than did Siat8 knockdown, because GalNAc-T can synthesize the gangliosides GD1a, GD1b, and GT1b, whereas Siat8 is not required for GD1a synthesis. Therefore, we next generated mutant P19 cells in which GalNAc-T was deleted using the CRISPR/Cas9 system. The CRISPR system is a bacterial adaptive immune system that uses an RNA-guided DNA nuclease to silence viral nucleic acids [25]. In comparison with TALEN-based genome editing, genome editing mediated by the CRISPR system provides a rapid and simple method to generate knockout or knock-in of a target gene in mammalian cells. Using this method, we successfully generated P19 cells deficient in the gangliosides targeted by BoNT/C without observing any off-target effects. In the knockout cells, H_C_/C was unable to bind and become internalized into the cells, and little cleavage of syntaxin-1 and SNAP-25 upon treatment with BoNT/C was observed. H_C_/C reportedly possesses two ganglioside-binding sites: GBP-2 and Sia-1 [13]. GD1a was able to bind GBP-2, but not Sia-1 in H_C_/C, and GD1b could bind Sia-1, but not GBP-2. In contrast, GT1b could bind to both GBP-2 and Sia-1. From our data, we conclude that any one of these three gangliosides can mediate the internalization of BoNT/C into neuronal cells, although the effect of GD1a was weak compared with those of GD1b or GT1b. Moreover, the presence of different kinds of gangliosides on the cell surface promotes the uptake of BoNT/C more efficiently. These observations largely confirm previous reports.

The present study primarily addresses evaluation of the utility of P19 cells to derive neurons for investigation of the intracellular mechanism of BoNT/C. Generally, transfecting primary cultured neurons or editing a target gene in these cells is known to be difficult. Therefore, the generation of knockout or transgenic mice is needed to prepare mutant neurons. We have now demonstrated the utility of P19 neurons for the investigation of the intracellular mechanism of BoNT/C, because neurons from P19 cells can be transfected with siRNA or used to generate knockout cells more easily than can cells from primary culture. In conclusion, genome-edited P19 cells generated by the CRISPR/Cas9 system can be used to efficiently identify and define the intracellular molecules involved in the toxic action of BoNTs.
